# Exploring the Impact of Reinforcing Filler Systems on Devulcanizate Composites

**DOI:** 10.3390/polym16111448

**Published:** 2024-05-21

**Authors:** Rounak Ghosh, Christian Mani, Roland Krafczyk, Rupert Schnell, Auke Talma, Anke Blume, Wilma K. Dierkes

**Affiliations:** 1 Sustainable Elastomer Systems, Elastomer Technology and Engineering, Department of Solids, Surfaces and Systems, Faculty of Engineering Technology, University of Twente, P.O. Box 217, 7500 AE Enschede, The Netherlands; ronforiit@gmail.com; 2 Evonik Operations GmbH, Paul-Baumann-Straße 1, 45772 Marl, Germany; christian.mani@evonik.com (C.M.); roland.krafczyk@evonik.com (R.K.); rupert.schnell@evonik.com (R.S.); 3 Elastomer Technology and Engineering, Department of Solids, Surfaces and Systems, Faculty of Engineering Technology, University of Twente, P.O. Box 217, 7500 AE Enschede, The Netherlands; a.g.talma-1@utwente.nl (A.T.);

**Keywords:** elastomer, devulcanizate, filler, composite, silane, silica, carbon black, sustainability, recycling

## Abstract

Composites revolutionize material performance, fostering innovation and efficiency in diverse sectors. Elastomer-based polymeric composites are crucial for applications requiring superior mechanical strength and durability. Widely applied in automotives, aerospace, construction, and consumer goods, they excel under extreme conditions. Composites based on recycled rubber, fortified with reinforcing fillers, represent a sustainable material innovation by repurposing discarded rubber. The integration of reinforcing agents enhances the strength and resilience of this composite, and the recycled polymeric matrix offers an eco-friendly alternative to virgin elastomers, reducing their environmental impact. Devulcanized rubber, with inherently lower mechanical properties than virgin rubber, requires enhancement of its quality for reuse in a circular economy: considerable amounts of recycled tire rubber can only be applied in new tires if the property profile comes close to the one of the virgin rubber. To achieve this, model passenger car tire and whole tire rubber granulates were transformed into elastomeric composites through optimized devulcanization and blending with additional fillers like carbon black and silica–silane. These fillers were chosen as they are commonly used in tire compounding, but they lose their reactivity during their service life and the devulcanization process. Incorporation of 20% (*w*/*w*) additional filler enhanced the strength of the devulcanizate composites by up to 15%. Additionally, increased silane concentration significantly further improved the tensile strength, Payne effect, and dispersion by enhancing the polymer–filler interaction through improved silanization. Higher silane concentrations reduced elongation at break and increased crosslink density, as it leads to a stable filler–polymer network. The optimal concentration of a silica–silane filler system for a devulcanizate was found to be 20% silica with 3% silane, showing the best property profile.

## 1. Introduction

The widespread use of elastomers across various applications raises environmental concerns related to waste management. Effectively addressing this challenge requires the efficient recycling and reuse of rubber [1]. However, a substantial obstacle to successful recycling is the crosslinked nature of elastomeric products. Vulcanization involves crosslinking polymer chains, creating a robust three-dimensional network. This alteration renders elastomeric material insoluble and infusible, presenting a challenge for effective recycling [2].

Devulcanization, the reversion of the vulcanization process, stands out as a sustainable advancement in elastomer recycling, distinguishing itself from conventional techniques such as grinding or regeneration, the mere replasticization of vulcanized elastomers [3,4]. Unlike these methods, which impose limitations on both quality and quantity for application, devulcanization introduces a more selective breakdown of the network. In contrast to the random breakdown of polymer networks in regeneration, devulcanization focuses on selectively dismantling crosslinks while preserving the polymer chains. This results in a higher tensile strength compared to reclaimed rubber obtained through alternative methods like grinding or regeneration [4,5,6,7,8,9,10].

Polymeric composites, particularly those involving elastomers, play a pivotal role in various industries due to their unique properties and versatility. Elastomeric composites show enhanced inherent qualities compared to their non-reinforced counterparts, providing improved mechanical strength, durability, and resistance to wear and tear [11,12,13,14]. These composites find extensive applications in automotive components, aerospace technology, construction materials, and consumer goods. The ability of elastomeric composites to withstand extreme temperatures, harsh chemicals, and dynamic stresses makes them indispensable in engineering solutions. Their lightweight nature contributes to energy efficiency in transportation, while their damping characteristics make them valuable for reducing vibrations. In essence, the significance of polymeric composites, especially elastomeric composites, lies in their capacity to revolutionize material performance across diverse sectors, fostering innovation and efficiency [15,16,17].

Recycled elastomeric composites, enriched with reinforcing fillers, epitomize sustainable material innovation. Harnessing the benefits of recycled rubber and integrating reinforcing agents like carbon black or silica enhances a composite’s strength and resilience. However, during the service life of a product and in the devulcanization process, fillers lose a part of their reinforcing strength. A way to mitigate this effect is to add fillers to the recycled rubber. 

This sustainable solution not only repurposes discarded elastomers, but also contributes to reduced environmental impact. Widely applicable in diverse industries, these recycled elastomeric composites containing reinforcing fillers offer a sustainable alternative to fossil-based materials, aligning with the growing emphasis on circular economy practices.

The utilization of devulcanized elastomers is hampered by their inferior properties compared to virgin elastomers. To address this limitation, the study aimed to narrow this gap by reinforcing the devulcanizate, transforming it into a composite based on an elastomeric devulcanizate. Model passenger car and whole tire rubber granulate were subjected to devulcanization through an optimized reference process. The quality of the devulcanizates was subsequently enhanced via the incorporation of reinforcing fillers. This investigation focuses on two key aspects: evaluating the influence of additional fillers on the devulcanizate and examining the impact of a coupling agent forming a polymer-filler bond on the devulcanizate composite properties.

## 2. Effect of Reinforcing Fillers on Devulcanizate Composites

In this study, both a model passenger car tire compound and whole tire (WT) rubber granulates were subjected to devulcanization under optimized process conditions and subsequently compounded using two different filler systems:

1. A highly dispersible silica (ULTRASIL^®^7000GR, Evonik Industries AG, Essen, Germany) together with a bifunctional silane (TESPD, bis[3-(triethoxysilyl)propyl]-disulfide) as a coupling agent between the filler and polymer was used. TESPD is a commonly used coupling agent for silica reinforcement of rubber, as it is able to split at the disulfide moiety. This results in two molecules comprising a 3-(triethoxysilyl)propyl] moiety, which can each attach to the silica. The sulfur moiety couples to the polymer.
2. A reinforcing-grade carbon black (N330).

These fillers were individually incorporated into the devulcanizate at four varying concentrations, while maintaining a consistent revulcanization formulation. The primary objective of this part of the study was to assess the impact of fillers on the resulting devulcanizate composite, particularly in terms of mechanical properties as well as filler–filler and filler–polymer interactions.

### 2.1. Material and Methods

**Figure 1:**
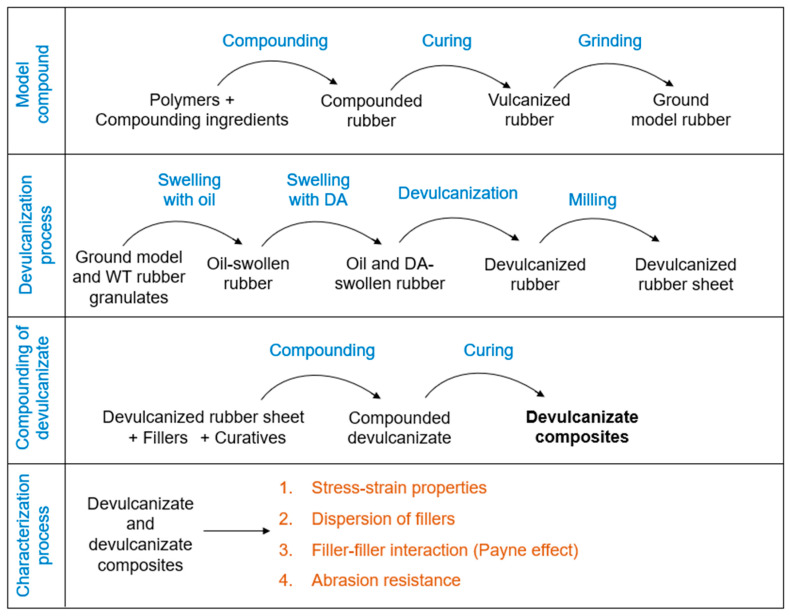
Experimental process of filler optimization for devulcanizate composites.

This section provides detailed information regarding the preparation of the feed materials, sampling plan, devulcanization, compounding with additional fillers, and the characterization processes. An overview of the experimental process is given in Figure 1.

#### 2.1.1. Model Compound Preparation

The formulation of a silica-filled model tire tread compound based on SBR/BR was developed for utilization as an input material in the devulcanization procedure. The various phases involved in the preparation of the model compound are described as follows.

##### Materials

The model tire tread formulation is given in Table 1.

##### Compounding

Compounding was carried out in two phases utilizing a 390 mL internal mixer (Model 350S) manufactured by Brabender GmbH & Co., Duisburg, Germany. In the initial stage, mixing commenced with a fill factor of 70%, an initial temperature of 80 °C, and a rotor speed set at 70 rpm. This initial mixing phase aimed to masticate the polymer and disperse the filler effectively, and enable the integration of other compounding ingredients. As the temperature rose, polymer viscosity decreased, while filler addition contributed to viscosity elevation. To optimize the silica–silane interaction, a temperature of 145 °C, known as the optimal reaction temperature, was chosen [19]. Consequently, adjustments were made to the initial temperature and rotor speed of the internal mixer to attain and uphold the desired temperature throughout the compounding process. The introduction of polymer, filler, and other compounding ingredients within the internal mixer led to a temperature rise from 80 °C to 145 °C due to high shear forces. Isothermal mixing and silanization were carried out at 145 °C for 5 min via rotor speed adjustments. The details of the mixing process are outlined in Table 2.

The masterbatch, which resulted from the initial mixing step, was allowed to rest at room temperature for one day before the final mixing process. Final mixing was conducted using a laboratory-scale, two-roll mill with a diameter of 9 cm, manufactured by Schwabenthan GmbH & Co., Berlin, Germany, and the curatives were incorporated at room temperature. The specific procedure is mentioned in Table 2. Following the final mixing, the compound was left to stand at room temperature for one day before the curing process.

##### Curing

The optimal cure time was determined using an RPA Elite, manufactured by TA Instruments, based in Delaware, OH, USA. Samples underwent curing at 160 °C following the T_95_ specification. Compression molding was conducted using an automatic press produced by Wickert Maschinenbau GmbH, Landau, Germany, employing molds sized at either 200 mm × 200 mm × 4 mm or 80 mm × 80 mm × 2 mm. Post-curing, the vulcanized sheets exhibited a tensile strength averaging around 16 ± 1 MPa, with an elongation at break of approximately 310 ± 30%.

##### Chopping and Grinding

The vulcanized sheets were chopped using a bale cutter. Pre-treatment for cryogenic grinding involved immersing the chopped samples in liquid nitrogen for 4–5 min to reach a temperature below the glass transition point, followed by grinding at room temperature. Grinding was executed utilizing a mechanical grinder manufactured by Fritsch, Germany, equipped with a 0.7 mm mesh screen.

#### 2.1.2. Preparation of WT Rubber Granulates

The WT rubber granulate used in this study was provided by Genan GmbH, Dorsten, Germany, and used without any further alteration.

#### 2.1.3. Swelling of Rubber Granulates

The ground model compound and WT rubber granulate underwent initial mixing with processing oil (TDAE), followed by subsequent mixing with the DA at room temperature. Each addition was followed by a one-day room temperature incubation period to allow for swelling. The kinetic viscosity of the TDAE oil was measured at 331 mm^2^/s at 40 °C and 18.4 mm^2^/s at 100 °C [20]. Due to the high viscosity of the TDAE oil, manual stirring was employed to mix the rubber granulate with the oil. Vinyl silane with peroxide (VP) was selected as the best DA based on the literature comparisons with sulphidic, amino, and mercapto silanes; thus, VP was utilized in this study [2]. The oil-swollen sample was subsequently re-swollen with VP and left for one day to allow for DA migration into the particles.

#### 2.1.4. Devulcanization of Rubber Granulates

The thermo-mechanical-chemical devulcanization process was conducted in a Plastograph EC internal mixer manufactured by Brabender GmbH & Co., Duisburg, Germany, featuring a mixing chamber volume of 50 cc; non-intermeshing, counter-rotating rotors; and a telescopic ram. To prevent oxidation at elevated temperatures, the cavity was sealed with paraffin wax to restrict air access. Devulcanization was carried out using the internal mixer set at a temperature of 180 °C, a rotor speed of 150 RPM, and a fill factor of 80%.

The same two-roll mill utilized in Section “Compounding” was employed for milling the devulcanized rubber at room temperature, employing a speed ratio of 1.25 and operating at 30 RPM. The nip gap was gradually reduced from 1 to 0.1 mm until the devulcanized rubber formed a band.

#### 2.1.5. Sampling Plan

The devulcanizates were prepared by using the optimum devulcanization conditions based on an earlier study [2]. The two types of devulcanizate were compounded separately with two filler systems with four different concentrations. The quantities of silane used as coupling agent were calculated according to Guy’s formula [18]. Considering the surface area of silica and functionality of the silane, 7.5% TESPT relative to the silica is sufficient for silanization under standard processing conditions. The sampling details of the filler optimization trials are given in Table 3.

It is important to note that the feedstock, the model compound as well as the whole tire granulate, already contain reinforcing fillers.

#### 2.1.6. Filler Compounding

The fillers were compounded with the devulcanizate using a Brabender Plastograph EC, Germany, with a 50 CC volume and 70% fill factor. The mixer was set to a temperature of 70 °C, which was increased to around 130 °C for silica compounding. The silica–silane compound was mixed for 3 min at an isothermally at 145 °C. For carbon black samples, the initial set temperature was 70 °C, but during compounding the temperature increased to around 120 °C; the compounding process, including mastication time, took a total of 6 min.

#### 2.1.7. Revulcanization

The devulcanized rubber samples were compounded using the formulation shown in Table 4 and was subsequently revulcanized.

The compounded devulcanized rubber underwent testing using the RPA 2000 Elite manufactured by TA Instruments, based in Delaware, USA at a temperature of 160 °C for a duration of 30 min, following the ASTM D7750-12 standard [21], to ascertain the optimal cure time. Sheets with a thickness of 2 mm were molded at 160 °C in accordance with the T_95_ specification using an automatic compression molding machine produced by Wickert Maschinenbau GmbH, Landau, Germany.

#### 2.1.8. Characterization Process

The characterization process contains four parts:

##### Stress–Strain Analysis

The stress–strain properties of the revulcanized rubber were assessed using a Z010 tensile tester manufactured by Zwick Roell GmbH & Co., Ulm, Germany, following the ASTM D412 standard [22]. Seven tensile dumbbells were tested for each sample, with the highest and lowest results omitted. The average of the remaining five values, along with error bars, are reported.

##### Dispersion

The macro-dispersion analysis of the silica within the compounded sample was conducted using a Dispergrader (optical microscope equipped with software to measure dispersion) manufactured by Alpha Technologies, based in the USA, under full-top light conditions at room temperature. Cylindrical samples measuring 25 cm in diameter and 12 mm in thickness were cut using a sharp knife, and the exposed cross-sections were examined. The filler distribution within the devulcanizate composites was analyzed based on the filler concentration.

##### Payne Effect

The Payne effect gives an indication of the micro-dispersion, the filler–filler interactions on a small scale, in the compounds. The analysis of the silica-filled devulcanizate composites was performed according to ASTM D8059-19 [23] in a rheometer manufactured by TA Instruments, USA. To measure the Payne effect of cured samples, the samples were revulcanized at 160 °C up to T_95_, and the Payne effect was measured at 60 °C in two strain sweeps, from low to high strains (from 0.33% to 14.1%) and from high to low strains (from 14.1% to 0.33%), for each sample. The curves from both sweeps were plotted, and the difference between the shear modulus at low strain and high strain of the low to high strain sweep was reported as the Payne effect.

##### Abrasion Resistance

The abrasion resistance of the rubber compounds was determined by the weight loss according to ASTM D5963 [24] on a rotary drum DIN abrasion tester manufactured by Montech, Switzerland.

### 2.2. Results

**Figure 2:**
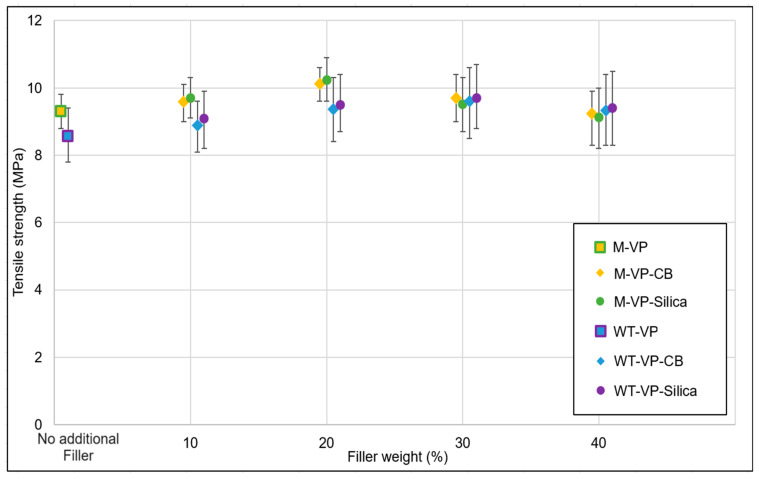
Filler optimization trials: tensile strength.

In a polymeric matrix, the filler system can create either a physical or a chemical interaction, depending on the type of filler: carbon fillers generate a physical interaction, while a silica–silane system forms a chemical bond with the polymer. As the concentration of filler increases, the mechanical strength of the elastomeric composite generally improves up to a certain point. However, if the polymer concentration is insufficient to interact with the further increased filler, it leads to a reduction in the overall mechanical properties of the system. This phenomenon is seen in Figure 2: The tensile strength of the de- and revulcanized elastomers increased with increasing filler concentration up to a certain level; above that limit reversal took place. For the model compound, the tensile strength reached around 10.3 MPa with 20% additional filler, and for WT granulate the maximum was around 9.7 MPa at 30% filler concentration. The samples filled with silica–silane demonstrated slightly higher values in comparison to carbon black. However, the average values of tensile strength follow a trend, which allows us to conclude that the silica-filled material performed, in general, better. Comparing the original and 20% additionally silica-filled model devulcanizate, a significant increase in tensile strength can be observed.

**Figure 3:**
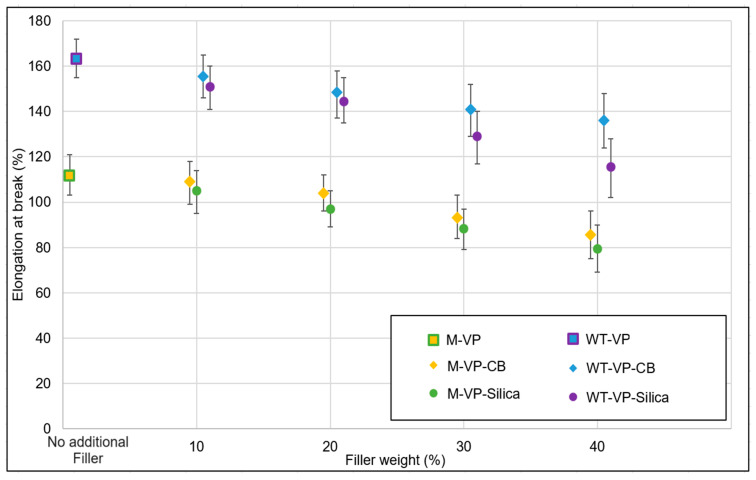
Filler optimization trials: elongation at break.

According to Figure 3, for both model compound and WT granulates, elongation at break decreased gradually with increasing filler concentration. For WT granulates, elongation at break was around 165% for the unfilled sample, and this was reduced to 118% for the 40% silica-filled samples and to 137% for the 40% carbon-black-filled samples. For the model compound, elongation at break was around 116% for original samples, which was reduced to 80% for 40% silica-filled samples and 83% for 40% carbon-black-filled samples.

**Figure 4:**
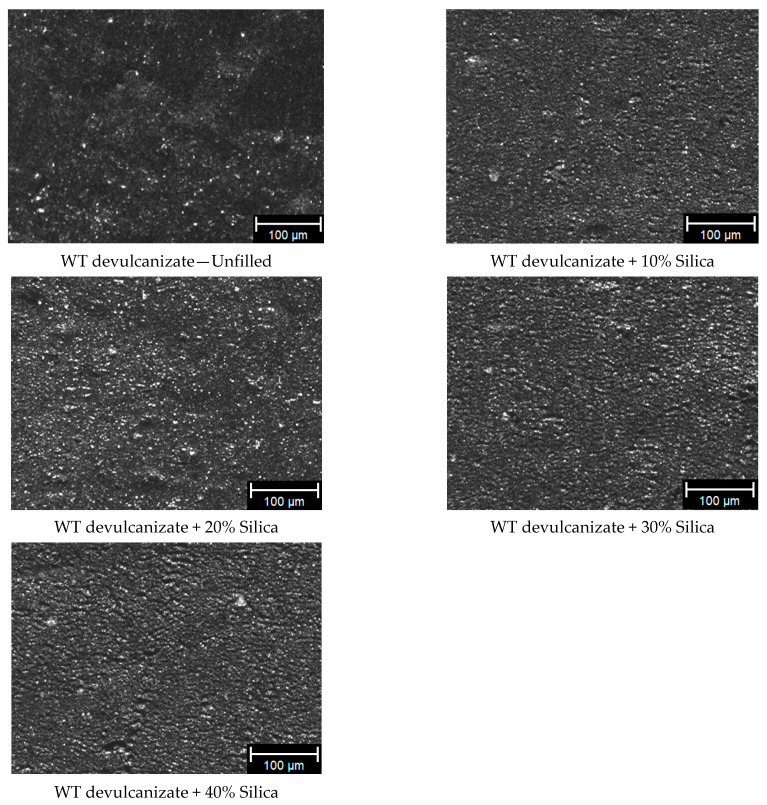
Filler optimization trials: dispersion measured using optical microscopy.

The dispersion analysis of the WT devulcanizate composites with increasing silica concentration is shown in Figure 4. With the increase in added filler concentration, an elevation in viscosity of the devulcanizate was observed, consequently leading to an increase in shear forces during the mixing process. These higher shear forces contribute to more effective mixing and, thus, enhance the dispersion of filler particles within the devulcanized matrix.

**Figure 5:**
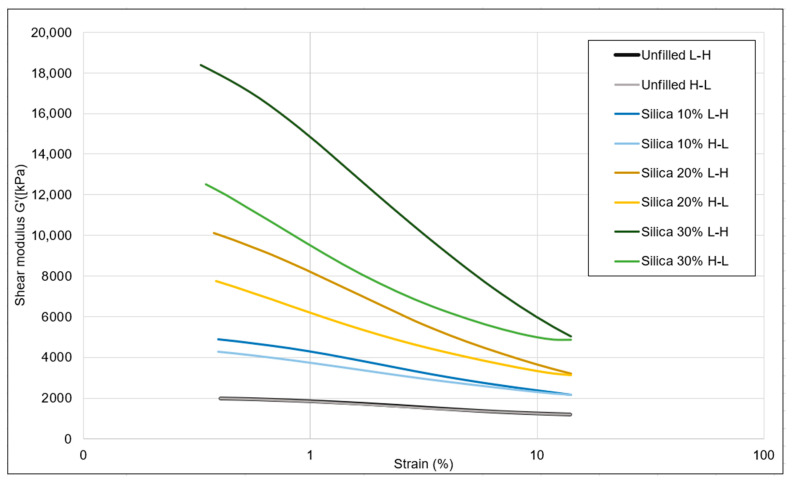
Filler optimization trials: Payne effect of the silica-filled samples.

Due to instrumental limitations (very high shear modulus), it was not possible to measure the Payne effect of the 40% filled samples. The notation L-H stands for the strain sweep from low to high, and H-L is the high to low sweep, as mentioned in the legend of Figure 5. With increasing silica concentration, the Payne effect increased gradually, which indicates higher filler interactions. Similarly, the difference between the shear modulus values of the two sweeps at low strains also increased. This can be explained by a change in the elastic behavior due to less matrix material with increasing concentrations of added filler and a stiffer matrix, resulting in a delay of the recombination of the filler at higher loadings, as shown in Table 5.

The devulcanizates without additional filler exhibited a minimal Payne effect, suggesting that the filler–filler interaction was already disrupted during devulcanization. The strain sweep conducted from low to high resulted in breaking of the remaining filler–filler network.

**Figure 6:**
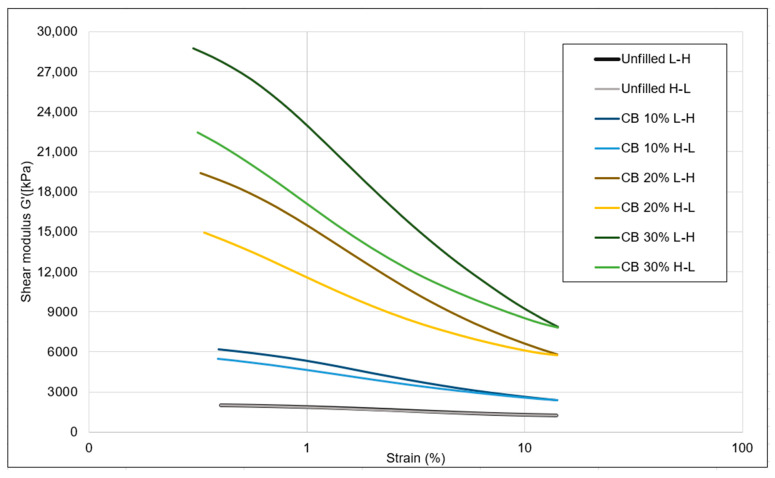
Filler optimization trials: Payne effect of the carbon-black-filled samples.

As shown in Figure 6 and Table 5, the carbon-black-filled samples showed the same trend in shear modulus as the silica-filled samples. The shear modulus values were higher for the carbon black samples compared to those of silica when compounded at the same weight percentage. This discrepancy in shear modulus is attributed to the higher volume of carbon black, as its density (1.8 g/cc) is lower than that of silica (2.5 g/cc). Consequently, the carbon black samples exhibited a comparatively elevated shear modulus.

Typically, abrasion resistance is quantified in terms of volume loss, but due to the density variations in the devulcanizate resulting from WT granulates, the abrasion resistance values were expressed in weight loss. The density of the model devulcanizate is constant within the sample as the feed material was uniform. However, for the whole tire (WT) devulcanizate, variations in density were observed, attributed to the diverse origins of granules from different tires and tire parts.

**Figure 7:**
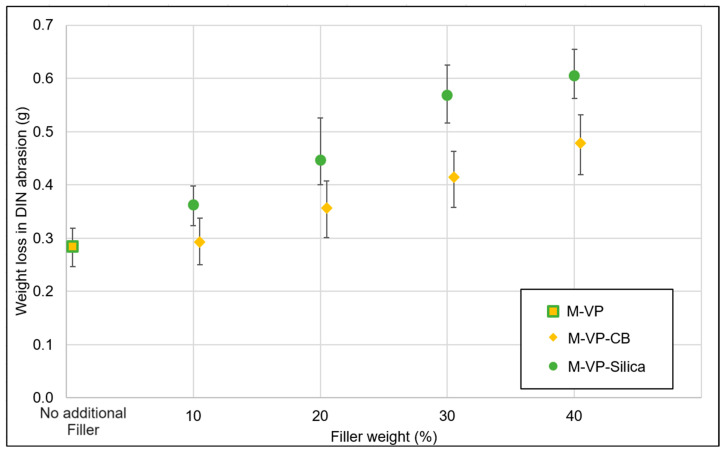
Filler optimization trials: abrasion.

According to Figure 7, the abrasion resistance of the sample with 10% additional carbon black was similar to the wear of the sample without any additional filler. With further increasing additional filler concentration, abrasion resistance decreased gradually, resulting in higher losses.

### 2.3. Discussion

The model compound for devulcanization contained 80 phr silica, a commonly used filler concentration; therefore, it does not have much capacity for a further filler loading. With increasing additional filler decreasing polymer content, the filler–polymer interaction was reduced due to insufficient polymer. The limit for this sample was 20%: above this additional filler loading, the tensile strength decreased for the model devulcanizates. WT rubber devulcanizates were also filled with additional silica or carbon black, and they showed a maximum tensile strength for 30% additional filler loading. Above this concentration the trend reversed. Compared to the model devulcanizate, higher elongation at break values were observed for the WT devulcanizates, probably due to the presence of natural rubber and carbon black in the feedstock.

With increasing filler concentration, the macro-dispersion, as measured using optical microscopy, improved due to increasing shear forces during blending of the compound with the additional filler.

During the Payne effect measurement within the first sweep, the shear modulus decreases with increasing strain, which indicates the breakdown of the filler network. During the second sweep from the high to low strain, the recombination of the filler network takes place resulting in an increase in shear modulus. With increasing filler concentration, the mobility of the filler in the matrix reduced, indicating less recombination for higher filler loadings. In the case of inadequate dispersion of fillers, the abrasion index is adversely affected leading to increased material loss during abrasion resistance testing.

The primary objective of these experiments was enhancing the mechanical strength of the devulcanizate composite through the inclusion of reinforcing fillers; this part of the study did not assess the network breakdown and miscibility analysis.

### 2.4. Conclusions

Compounding of around 20% of additional filler can improve the tensile strength of the devulcanizate composites by up to 15%. In addition to the filler cost, a sacrifice in elongation at break and abrasion resistance was noticed.

In comparison to samples filled with silica, those filled with carbon black did exhibit significant improvements in mechanical or viscoelastic properties. Therefore, further optimization on the silica–silane filler system was performed in order to elaborate the best filler-coupling agent ratio. In addition, silica is a widely used in passenger car tires due to the enhancement in fuel efficiency of a vehicle.

## 3. Effect of Silanes on Devulcanizate Composite Properties

In this investigation, the silane concentration relative to the silica concentration was varied. In general, a fixed ratio of silica to silane is used; however, in the devulcanizate, carbon black as well as silica were already present in the feed material. Carbon black might interfere with the silanization of the additional filler by adsorbing and, thus, deactivating the silane, and the originally contained silica might become activated and, Thus, consume some of the added silane.

The devulcanizate was compounded with additional silica and varying amounts of silane. Highly dispersible silica (ULTRASIL^®^ 7000GR) and TESPD were compounded in six different combinations, keeping the same revulcanizate formulation.

### 3.1. Experimental Process

**Figure 8:**
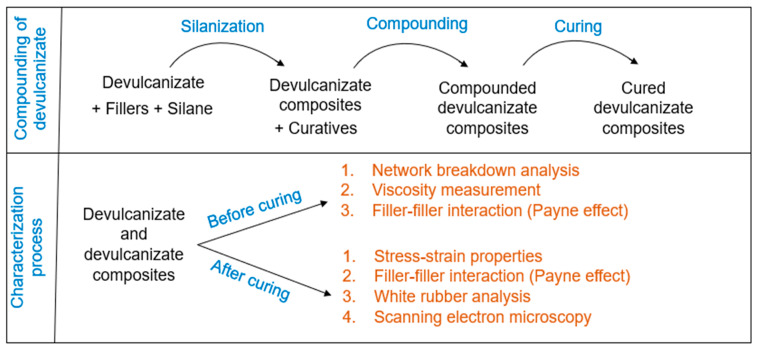
Experimental flowchart for silanization reactions on devulcanizates and devulcanizate composites.

The preparation of the feed devulcanizate, sampling plan, and characterization processes are described in this section and illustrated in Figure 8. 

#### 3.1.1. Preparation of WT Devulcanizate

Preparation of the WT granulates was performed according to the same procedure described in Section 2.1.2, Section 2.1.3, Section 2.1.4 and Section 2.1.6.

**Figure 9:**
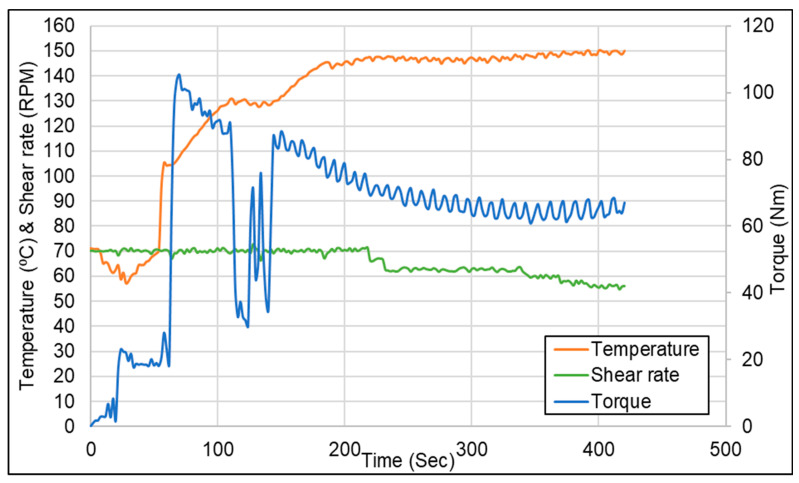
Compounding curve for the silanization reaction.

The mixing curve, illustrated in Figure 9, shows the mastication of the devulcanizate for 1 min, addition of silica for 1 min, ram cleaning and replacement of overflown silica for 30–45 s, and finally the silanization reaction during isothermal mixing for 220 to 250 s, depending on the time needed for returning escaped silica into the mixing chamber [19].

#### 3.1.2. Sampling for Silanization Trials

A total of 20% silica with 1.5% silane was reported as the optimal filler concentration in Section 2.3; the quantity of silane coupling agent was calculated according to Guy et al. [18]. To evaluate the effect of silanization, samples without silica and silane were used as references, and the silane concentration was varied while keeping the silica concentration constant. The sample without additional silica but with additional silane was tested to assess the reactivation of silica present in the feed material in the devulcanizate composite. The sampling details of the silanization trials are given in Table 6.

All samples were devulcanized while maintaining the same procedure as described in Section 2.1.4. They were revulcanized using the same formulation mentioned in Section 2.1.7.

#### 3.1.3. Characterization Process

The characterization process consists of six parts:

Stress–Strain Properties in Tensile Mode—Described in Section “Stress–Strain Analysis”.

Filler Interaction by Payne Effect Analysis—Described in Section “Stress–Strain Analysis”.

##### Degree of Network Breakdown by the Horikx Verbruggen Plot

The evaluation of crosslink density, as outlined in ASTM D 6814-02 [25], employs the equilibrium volume swelling method and the Flory–Rehner equation [26]. Initially, samples undergo extraction in acetone to eliminate polar components, followed by drying. Subsequently, extraction with tetrahydrofuran removes non-polar parts. The percentage of network breakdown was calculated using Equation (1) [27,28]:

(1) $$Network\;breakdown\;percentage\left(\%\right)=\frac{V_{c1}-V_{c2}}{V_{c2}}.$$

In this equation, V_c1_ and V_c2_ represent the crosslink densities of the samples before and after devulcanization, respectively [26,27,28]. It is worth noting that the crosslink density determined using the Flory–Rehner equation may not reflect the true value in a filled compound. To ascertain the precise crosslink density, the Kraus correction was employed.

(2) $$V_{actual}=\frac{V_{apparent}}{1+k\times \Phi }$$

(3) $$\Phi =\frac{Weight\;fraction\;of\;the\;filler\times density\;of\;the\;compound\times W_{b}}{Density\;of\;the\;filler\times W_{a}}$$

Here, V_apparent_ represents the measured crosslink density as determined using the Flory–Rehner equation, while V_actual_ denotes the actual crosslink density after adjustment for the filler. K stands as a constant specific to the filler employed, Φ signifies the volume fraction of the filler in the specimen, W_b_ indicates the weight of the specimen prior to extraction, and W_a_ represents the weight of the specimen post-extraction of all soluble components, including the polymer sol fraction, oil, and soluble chemical residues [27,28].

The thresholds for random scission and crosslink scission were determined by assessing the sol content of the feed material utilizing the Horikx–Verbruggen method [28]. By plotting the sol fraction and network breakdown values of the devulcanizates on a graph, the nature of network breakdown can be inferred. For each data point of network breakdown versus sol content, the average outcome of five samples was computed.

**Figure 10:**
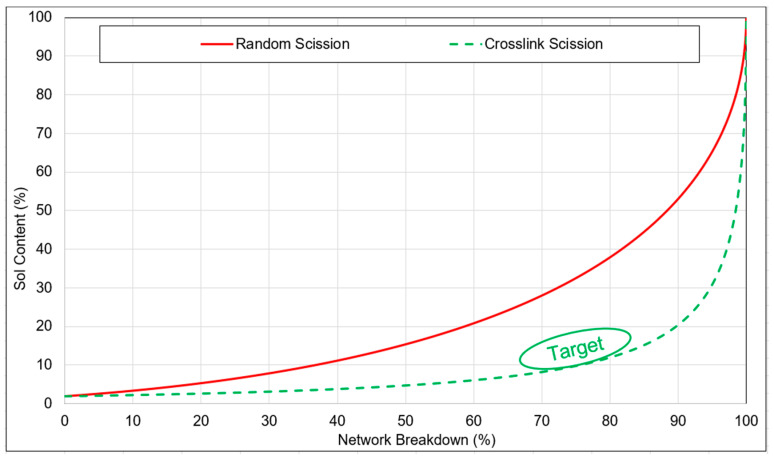
Sol content versus network breakdown percentage for random and crosslink scission according to the Horikx–Verbruggen plot [2].

In Figure 10, the highlighted green zone represents the desired range for the devulcanized rubber, aiming for the optimal balance between the minimal sol fraction and maximum devulcanization percentage. Attaining higher values necessitates the breaking of monosulphidic bonds, which correlates with an increased degree of random scission. Additionally, it is important to note that bound rubber cannot be dissolved, thereby restricting the sol content.

##### Homogeneity Evaluation by White Rubber Analysis

Following the devulcanization process, residual undevulcanized particle cores may persist within the devulcanizate. While devulcanized polymer chains exhibit homogenous miscibility with compatible polymers or compounds, undevulcanized particle cores do not share the same property. Consequently, it becomes crucial to evaluate the quantity, dimensions, and overall area of these undevulcanized particle cores. To facilitate this analysis, the white rubber analysis (WRA) method was devised.

In this quantitative analytical approach, the devulcanizate was blended with a bright-white, polybutadiene-based compound incorporating titanium dioxide as a colorant. The selection of a white colorant was deliberate, enhancing the contrast between the background and devulcanizate, thus facilitating the quantitative characterization process. Samples were prepared by incorporating 10% devulcanizate into this white rubber compound, resulting in a gray compound where any remaining undevulcanized particles were discernible as brownish spots. Digital analysis of the particles and their size distribution was conducted using a VHX 5000 digital microscope manufactured by Keyence.

##### Processability of the Rubber in Terms of Viscosity

The viscosities samples were determined using an MV 2000 VS viscometer supplied by Alpha Technologies GmbH, Bellingham, WA, USA, following the guidelines specified in the ASTM D1646-19a standard [29]. In this testing procedure, a large rotor (ML) with a diameter of 38.1 mm was employed, and the test temperature was maintained at 100 °C. A fixed sample weight of 20 g was pre-heated for one minute, and the viscosity, recorded in Mooney units (MU), was obtained after another 4 min duration.

##### Scanning Electron Microscopy (SEM)

SEM analysis was performed using a TableTop SEM PhenomXL manufactured by ThermoFisher, Waltham, MA, USA. The cross-section and fracture surfaces of all the samples were analyzed to evaluate the dispersion of the fillers with variable silane concentrations. The experimental conditions were kept the same for all measurements: voltage: 5 kV, beam intensity: image, detector: BSD full, vacuum: low (60 Pa), averaging: high, and scan size: 3840 × 2400.

### 3.2. Results and Discussion

**Figure 11:**
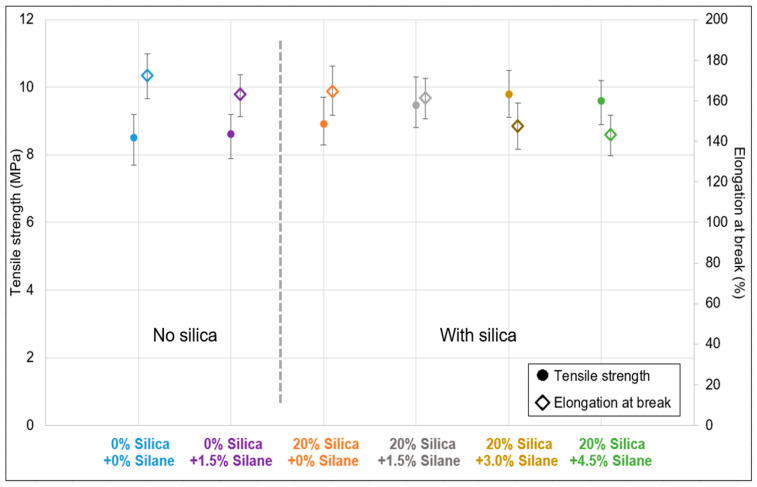
Silanization trials: stress–strain properties.

Figure 11 displays the stress–strain characteristics. The sample without additional silica and silane exhibited the lowest tensile strength and the highest elongation at break. When only 1.5% silane was added, no significant increase in tensile strength was noticed but a slight reduction in elongation at break was noticed. In the case of the additionally filled samples, the silica concentration remained constant at 20% (*w*/*w*), while the silane concentration was varied from no silane to 4.5% (*w*/*w*); the commonly used concentration is 1.5% (*w*/*w*). In this series, the tensile strength improved up to a silane concentration of 3%, beyond which no further enhancement was observed. Elongation at break gradually decreased as the silane concentration increased due to the formation of an additional filler–polymer and polymer–polymer network during the revulcanization process. This trend was expected, as stronger materials, in general, show reduced strain.

**Figure 12:**
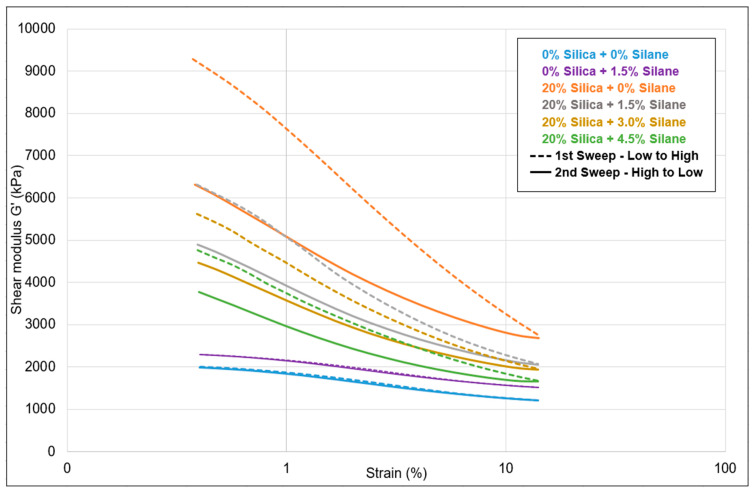
Silanization trials: Payne effect.

Figure 12 presents a comparative analysis of the Payne effect for not filled and silica-filled samples with varying silane concentrations. The strain sweep curves represented by dotted lines depict the sweep from low to high strain, during which the filler network is broken, leading to a decrease in shear modulus. Conversely, the solid curves represent the high to low strain sweep, during which recombination of the filler network occurs, resulting in an increase in the shear modulus.

For the samples without additional silica (blue and violet), both sweeps show low shear moduli (Table 7), indicating effective dispersion of the filler. Upon the addition of silica, in the difference between the shear moduli in the first sweep of low to high strain, the Payne effect increased. This indicates stronger and more frequent interactions between the fillers. This is a commonly seen effect: as the number of silica particles increases, their distance decreases and filler–filler interaction becomes more probable. As the silane concentration increased, the Payne effect gradually decreased, indicating better dispersion of the filler with less filler–filler interactions: with increasing silane concentration, the degree of silanization increases; see Table 7. This increases the compatibility of the silica with the rather apolar polymers and, thus, enhances the filler–polymer interaction.

**Figure 13:**
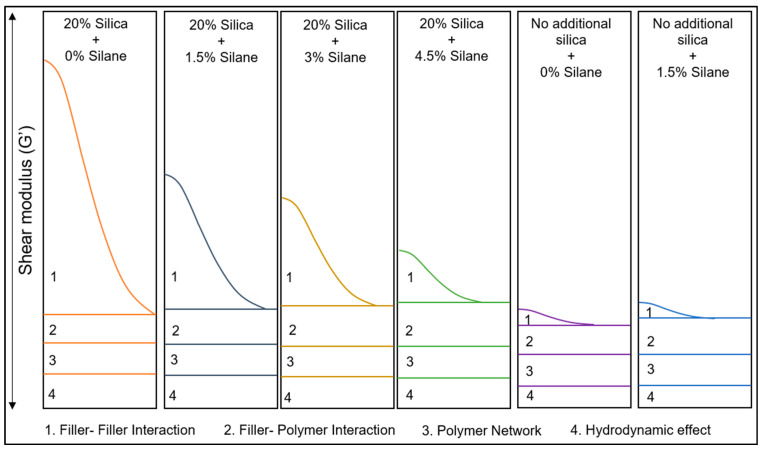
Silanization trials: schematic of different components of the Payne effect.

The Payne effect curves are a product of four variable effects, as illustrated in Figure 13: filler–filler interaction, filler–polymer interaction, polymer network, and the hydrodynamic effect. For samples without additional silica but containing a certain amount of silica from the original compounding, the only parameter that changes is the filler–polymer interaction: filler–polymer bonds might be broken in the mixing process these samples undergo. The other three parameters remain constant in this case. Conversely, in samples filled with additional silica, an increase in silane concentration results in an increase in filler–polymer interaction and a gradual decrease in filler–filler interaction due to the compatibilization and coupling of the silica by the silane. The polymer–polymer network remains unchanged. There might be a slight reduction in the hydrodynamic effect due to the addition of an excess of liquid silane, which did not react with silica.

Based on the findings from the Payne effect data, no reduction was observed when silane was introduced to the samples that were not additionally filled. The consistent Payne effect values indicate the absence of silica reactivation: If silane had reacted with silica, a lower Payne effect would have been anticipated. The lack of any alteration in the Payne effect values confirms the absence of silica reactivation.

**Figure 14:**
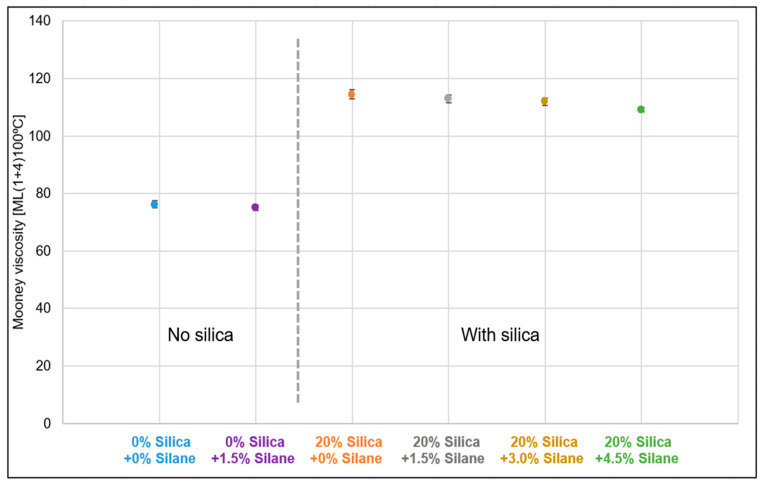
Silanization trials: viscosity.

Figure 14 illustrates a comparative analysis of the viscosities of the samples with and without additional silica and different silane concentrations. The viscosity of the samples without additional silica was observed to be in the range of 75 MU to 78 MU. Upon adding 20% (*w*/*w*) silica, the viscosity increased to approximately 115 MU. The viscosity gradually decreased with increasing silane concentration, reaching around 110 MU. As the silica concentration was constant, an excess of silane contributed to the plasticization effect.

**Figure 15:**
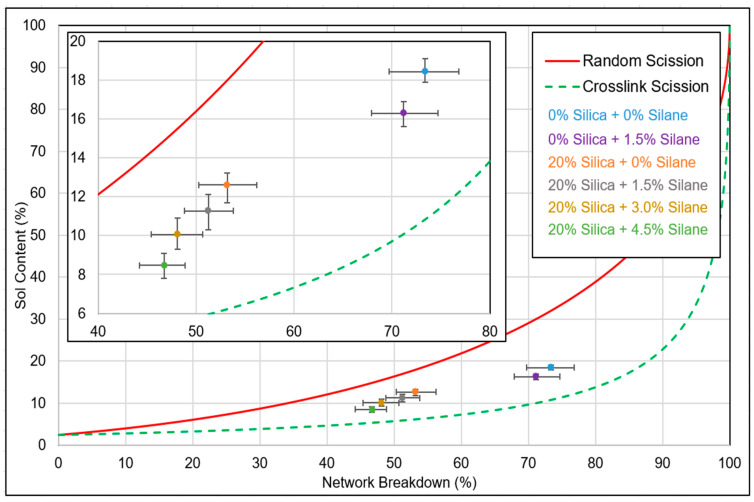
Silanization trials: network breakdown.

Figure 15 depicts a comparative Horikx–Verbruggen analysis of samples with and without additional silica with varying silane concentrations. The red line represents the limit of random scission, while the dotted green line represents the limit of crosslink scission. The average values of five samples are plotted in terms of network breakdown and sol content. All samples underwent the same devulcanization process, followed by separate compounding with silica and silane.

The unfilled samples exhibited a network breakdown ranging from 70% to 75%. Upon the addition of 20% (*w*/*w*) silica, when the silane concentration was varied from 0% to 4.5%, the network breakdown percentage decreased from 55% to 45%. The variation in network breakdown was attributed to the increase in silane concentration, as this resulted in an additional polymer–filler network, which counterbalances the reduction in crosslink density in the devulcanization process. Though a Krauss correction for the presence of fillers was performed, the reduction in network breakdown percentage could be due to the fact that the additional 20% silica forms a different type of reinforcement for which the Krauss correction has limited applicability. With increasing silane concentration, the interaction between the filler and the polymer improved due to the silanization reaction, leading to an additional network and, consequently, lower net network breakdown.

**Figure 16:**
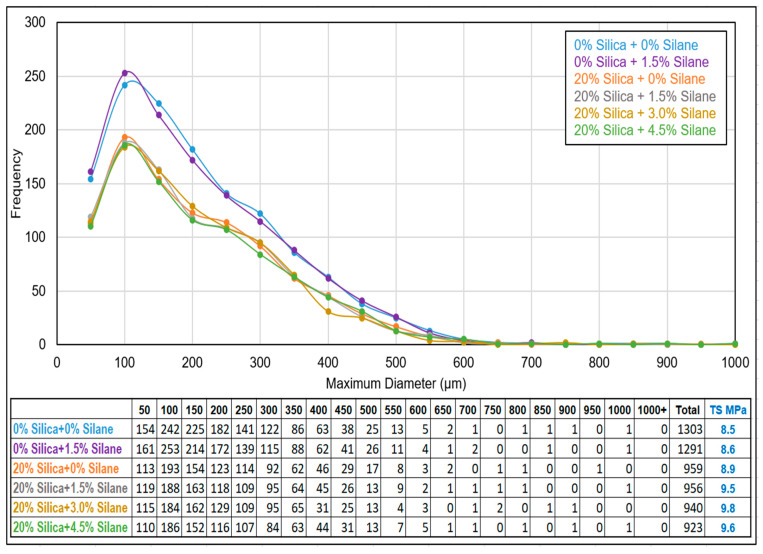
Silanization trials: white rubber analysis.

**Figure 17:**
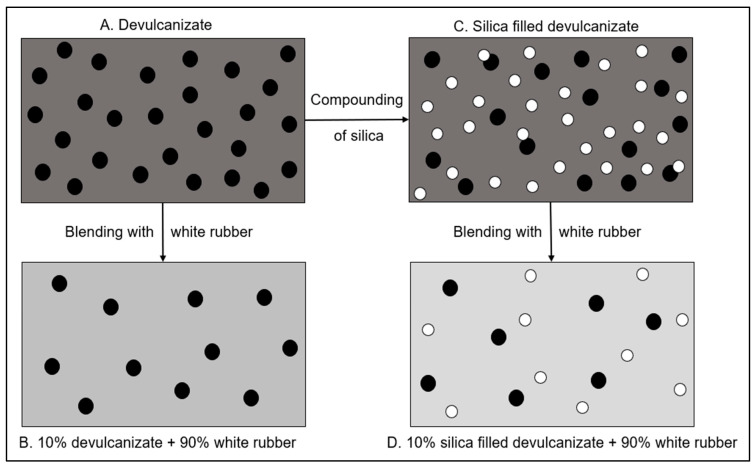
Schematic diagram of white rubber analysis: white rubber samples containing vulcanizates with and without additional silica.

According to Figure 16, samples without silica exhibited a total number of visible undevulcanized particles in the range of 1290–1300. However, with the addition of 20% (*w*/*w*) silica together with varying amounts of silane, the total number of visible particles decreased to approximately 920–960. The presence of silica in the additionally filled composite samples led to a decrease in the total number of immiscible particles compared to not-filled devulcanizate, as shown in Figure 17. Due to the contribution of additional silica, the relative volume of undevulcanized particles was reduced, resulting in a lower number of visible particles.

**Figure 18:**
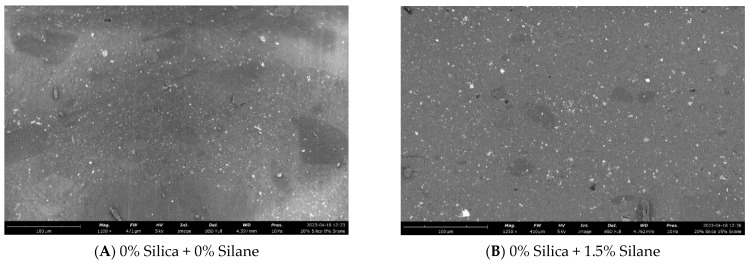

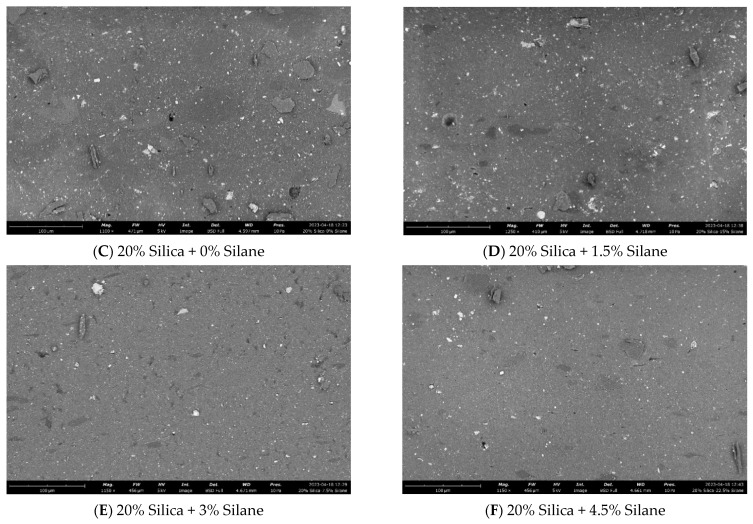
Silanization trials: macro-dispersion (cross section analysis by SEM).

According to the observations in Figure 18, an increase in silane concentration resulted in improved dispersion of silica: Figure 18C,D showed comparatively poor dispersion compared to Figure 18E,F, which correlates with the Payne effect values.

**Figure 19:**
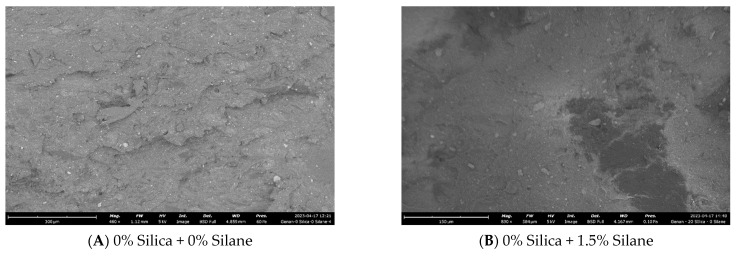

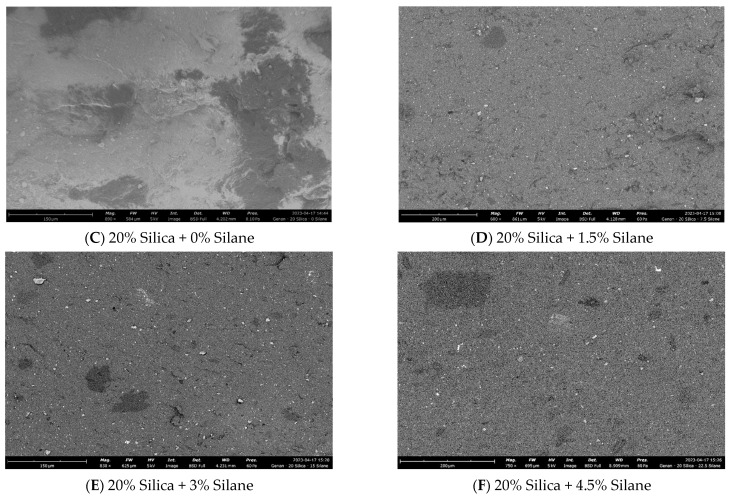
Silanization trials: fracture surface analysis via SEM.

Based on the findings in Figure 19, in which fracture surfaces are illustrated, the material exhibited smoother fracture surfaces as the silane concentration increased compared to no-silane samples. With increasing silane concentration, the silica dispersion became more homogeneous. Fractures are generated from defective spots; therefore, a more homogeneous and uniform distribution will cause less defects, leading to a smoother fracture surface and resulting in better tensile properties, as seen in Figure 11.

### 3.3. Conclusions

With additional 20% (*w*/*w*) silica and increasing silane concentration, the tensile strength, Payne effect, and dispersion improved due to better polymer–filler interactions following a higher degree of silanization. With increasing silane concentration, elongation at break and net network breakdown were reduced due an increase in crosslink density by the formation of short, stable filler–polymer bonds during the silanization reaction. Viscosity was reduced due to the plasticization effect of silanes.

A total of 20% (*w*/*w*) silica with 3% (*w*/*w*) silane showed the best properties in terms of mechanical strength; beyond this concentration, no further improvement was noticed. This can be considered as the optimum concentration for the addition of a silica–silane filler system to the devulcanizate composite.

This high-quality devulcanized composite can be partially integrated into new high-performance rubber products, such as tires and conveyor belts. It can also be used to create lower tensile strength products like road underpads and acoustic protection materials, which can be made entirely from the devulcanized composite.

## Figures and Tables

**Table 1:** Compounding formulation of the model tire tread compound.

Function	Ingredient	Trade Name	Supplier	Quantity (phr)
Polymer	SSBR ^1^	Sprintan 4601	Trinseo (Wayne, PA, USA)	70
	BR ^2^	CB 24	Arlanxeo (Hague, The Netherlands)	30
Filler system	Silica	ULTRASIL^®^ 7000 GR	Evonik (Essen, Germany)	80
	Silane	Si 266^®^	Evonik (Essen, Germany)	5.8 *
Activators	Zinc oxide	Merck Zinc Oxide	Sigma-Aldrich (St. Louis, MI, USA)	3
	Stearic acid	Merck Stearic Acid	Sigma-Aldrich (St. Louis, MI, USA)	2
Plasticizer	TDAE oil ^3^	Vivatec	H&R (Houston, TX, USA)	25
Curatives	Sulphur	Merck Sulphur	Sigma-Aldrich (St. Louis, MI, USA)	1.5
	Primary accelerator	Santocure CBS	Flexsys (Drive Akron, OH, USA)	1.7
	Secondary accelerator	Perkacit DPG	Flexsys (Drive Akron, OH, USA)	2.5

^1^. Solution-polymerized styrene butadiene rubber; ^2^. Polybutadiene rubber; ^3^. Treated distillated aromatic extract. * Silane amount was calculated according to the formula of Guy et al. [18].

**Table 2:** Mixing process of the model tire tread compound.

Masterbatch (First) Step		Final (Second) Step	
Action	Time [mm:ss]	Action	Time [mm:ss]
Polymer	00:00–00:30	Masterbatch	-
Mastication	00:30–01:30		
½ (Silica + silane)	01:30–02:00	Mixing	00:00–02:00
Mixing	02:00–03:00		
½ (Silica + silane) + additives	03:00–03:30	Curatives	02:00–02:30
Mixing (140–150 °C)	03:30–04:30		
Ram sweep	04:30–05:00	Mixing	02:30–09:00
Mixing (target 145 °C)	05:00–09:00		
Discharge and sheeting	-	Discharge	-

**Table 3:** Sampling plan for filler optimization trials.

Devulcanizate	Abbreviations	Silica + Silane (*w*/*w* %)	Carbon Black (CB)
Model—VP	M-VP	1. 10% Silica + 0.75% Silane	1. 10% CB
WT granulates—VP	WT-VP	2. 20% Silica + 1.50% Silane	2. 20% CB
		3. 30% Silica + 2.25% Silane	3. 30% CB
		4. 40% Silica + 3.00% Silane	4. 40% CB

**Table 4:** Revulcanization formulation.

Function	Component	Weight (%)
Base polymer	Devulcanized rubber sample	100
Activators	Zinc oxide	4
	Stearic acid	2
Curing aid	Sulphur	2
Accelerator	CBS	1

**Table 5:** Payne effect values for filler optimization trials.

Filler Type	Filler Quantity (*w*/*w* %)	Low to High Strain Sweep: Shear Modulus G’ (KPa)		High to Low Strain Sweep: Shear Modulus G’ (KPa)		Payne Effect (KPa) ^#^
		Max	Min	Min	Max	
Unfilled	-	1997	1212	1211	1987	785
Silica–silane	10	4867	2163	2147	4286	2704
	20	10,131	3191	3146	7745	6940
	30	18,392	5012	4875	12,512	13,380
Carbon black	10	6193	2369	2357	5465	3824
	20	19,400	5794	5724	14,954	13,606
	30	28,724	7878	7789	22,440	20,846

^#^ Payne effect = difference between the maximum and minimum shear modulus of the low to high strain sweep.

**Table 6:** Sampling plan of silanization trials.

Sample	Compounding Details	
	Silica (*w*/*w*%)	Silane (*w*/*w*%)
1	0	0
2	0	1.5
3	20	0
4	20	1.5
5	20	3.0
6	20	4.5

Feed material: WT granulates; temperature: 180 °C; residence time: 6 min; shear rate: 150 RPM; fill factor: 80%; VP concentration: 5%.

**Table 7:** Silanization trial: Payne effect data—shear modus.

Sample Details	Low to High Strain Sweep: Shear Modulus G’ (KPa)		High to Low Strain Sweep: Shear Modulus G’ (KPa)		Payne Effect ^#^ (KPa)
	Max	Min	Max	Min	
0% Silica + 0% Silane	1997	1212	1211	1987	785
0% Silica + 1.5% Silane	2297	1512	1511	2287	785
20% Silica + 0% Silane	9290	2747	2674	6317	6543
20% Silica + 1.5% Silane	6308	2073	2056	4898	4235
20% Silica + 3% Silane	5612	1953	1937	4469	3659
20% Silica + 4.5% Silane	4752	1675	1658	3776	3077

^#^ Payne effect = difference between the maximum and minimum shear modulus of the low to high strain sweep.

## Data Availability

Data are contained within the article.
